# Plant Heterotrophic Cultures: No Food, No Growth

**DOI:** 10.3390/plants13020277

**Published:** 2024-01-17

**Authors:** Roman K. Puzanskiy, Daria A. Romanyuk, Anastasia A. Kirpichnikova, Vladislav V. Yemelyanov, Maria F. Shishova

**Affiliations:** 1 Laboratory of Analytical Phytochemistry, Komarov Botanical Institute of the Russian Academy of Sciences, 197022 St. Petersburg, Russia; puzansky@binran.ru; 2 Laboratory of Genetics of Plant-Microbe Interactions, All-Russia Research Institute for Agricultural Microbiology, 196608 St. Petersburg, Russia; d.romanyuk@arriam.ru; 3 Faculty of Biology, St. Petersburg State University, 199034 St. Petersburg, Russia; nastin1972@mail.ru (A.A.K.); bootika@mail.ru (V.V.Y.)

**Keywords:** plant suspension culture, plant heterotrophic culture, sucrose, organic uptake, central metabolism, starvation, expansion growth

## Abstract

Plant cells are capable of uptaking exogenous organic substances. This inherited trait allows the development of heterotrophic cell cultures in various plants. The most common of them are *Nicotiana tabacum* and *Arabidopsis thaliana*. Plant cells are widely used in academic studies and as factories for valuable substance production. The repertoire of compounds supporting the heterotrophic growth of plant cells is limited. The best growth of cultures is ensured by oligosaccharides and their cleavage products. Primarily, these are sucrose, raffinose, glucose and fructose. Other molecules such as glycerol, carbonic acids, starch, and mannitol have the ability to support growth occasionally, or in combination with another substrate. Culture growth is accompanied by processes of specialization, such as elongation growth. This determines the pattern of the carbon budget. Culture ageing is closely linked to substrate depletion, changes in medium composition, and cell physiological rearrangements. A lack of substrate leads to starvation, which results in a decrease in physiological activity and the mobilization of resources, and finally in the loss of viability. The cause of the instability of cultivated cells may be the non-optimal metabolism under cultural conditions or the insufficiency of internal regulation.

## 1. Introduction

Higher plants are evolved from green algae, which have a flexible system of trophic adaptation to environmental conditions based on their ability to photosynthesize and to assimilate a wide range of organic compounds [1,2]. This trophic plasticity, inherited by the cells of higher plants, contributed to the emergence of a complex differentiated plant organism consisting of both photoautotrophic “source” tissues and heterotrophic “sink” tissues. Photosynthesis in higher plants is mostly performed in aboveground organs. This process mainly takes place in leaf parenchyma cells, being carbon donors for the whole plant [3]. However, a number of non-photosynthetic organs and tissues also belong to the shoot, including generative organs that have partially or completely lost their ability to photosynthesize. Heterotrophic tissues also include tissues specializing in various functions, such as transport, storage, etc. The ability of the cells of these organs and tissues to uptake carbon from external space ensures their vital activity and growth. The role of heterotrophic nutrition in plant cells changes during development. Firstly, dependence on organic carbon supply is clearly manifested during germination and the early stages of development, before the formation of the functional photosynthetic apparatus [4]. Secondly, during further development, it is possible to identify diurnal [5,6] and seasonal rhythms [7] that determine the accumulation and consumption of storage. Thirdly, the heterotrophic nutrition of cells is clearly demonstrated during the reutilization of carbon and nitrogen from senescent organs [8].

Thus, even multicellular plant organisms, defined as photoautotrophs, retain trophic plasticity potential. Heterotrophic nutrition provides plants with the opportunity to expand into ecological niches where photosynthesis is limited by a lack of light or CO_2_. There are various ways to obtain exogenous organic compounds from plants [1,2]. One of them is parasitism, which is quite widespread among plants. There are more than 4500 species of vascular plants from 28 families capable of parasitism and affecting other plants. Among them are hemiparasites, which retain the photosynthetic apparatus, and fully heterotrophic holoparasites. Plant parasitism can be both obligate and facultative [9,10,11]. Plants have the ability to use organic compounds produced by mycorrhizal fungi. Such a manner of nutrition is known as mycoheterotrophy, and can be both total and partial. The plant can be mycoheterotrophic throughout the entire life cycle, or only during germination [12]. In addition, the ability of plants to absorb organic nutrients, especially amino acids, directly from the soil is also identified [13].

The capacity of plants, their organs, tissues, and individual cells to live off an external carbon source is used to support their growth and development in vitro. Cultures of isolated plant organs and tissues began to be obtained from the beginning of the 20th century. The discovery of the value of auxin and its synthetic analogues, as well as the role of B vitamins, made it possible to obtain the first callus cultures by the end of the 1930s. In the second half of the 1950s, with the discovery of another phytohormone—kinetin (and its natural analogues)—the formation of the concept of hormonal growth control began [14]. In the early 1960s, Toshio Murashige and Folke Skoog [15] developed the composition of culture medium, which is widely used to this day, despite a large number of modifications being needed depending on the type of cells and the ultimate goal of culture maintenance. From that moment, the modern age of the intense use of plant cell cultures began. Callus cultures are represented by conglomerates of undifferentiated cells [16]. In the mid-1950s, the first suspension cultures were obtained based on calli as a result of resuspension. Suspension cultures are maintained under constant agitation [17]. They have a number of advantages, which are greater cell homogeneity, a shorter cultivation cycle, and a higher growth rate compared to calli. In addition, suspension cultures are easier to scale [17,18].

Cell cultures are maintained under aseptic controlled conditions [19]. This allows a high reproducibility of results to be achieved and the use cell cultures as model systems for studying many processes, like cell specialization [20], organogenesis [21], embryogenesis [22], reproductive processes, cellular responses to endogenous [20,23,24] and exogenous factors [25,26,27], programmed cell death [28], the metabolome [29], etc. In addition, due to their advantages, cell cultures have found wide application in biotechnology [17,30,31,32,33]. Plant cell cultures, including genetically engineered ones, are used for the production of biologically active secondary compounds, including alkaloids, terpenoids, phenylpropanoids, etc. [33,34,35,36,37,38]. They are applied for heterologous protein production [18,33,39,40]. Potentially, plant cells can be used in the food industry as producers of healthy food rich in useful compounds, protein, and fibers [41,42]. Plant cultures are used for cryopreservation and vegetative reproduction [43,44].

Over recent decades, cells of a large number of plants, mainly angiosperms and mosses, have been introduced into culture [17,31,45,46,47]. Among them, cell cultures of *Arabidopsis thaliana* [34] and *Nicotiana tabacum* [48] have acquired the greatest importance as model objects. The interest in *A. thaliana* cell cultures is not surprising due to the fact that this plant is the most common model object of modern plant biology. Its genome is sequenced and well annotated, and various databases containing information on transcription profiles, protein diversity, metabolic pathways, etc., have been developed [49,50,51]. BY-2 tobacco cell line application has been going on for more than 50 years [52]. The peculiarity of this line is its very rapid growth and ability to synchronize the cell cycle [23,53,54]. Cell lines based on BY-2 have been very actively used in the development of modern plant biotechnology [39,55]. 

**Figure 1:**
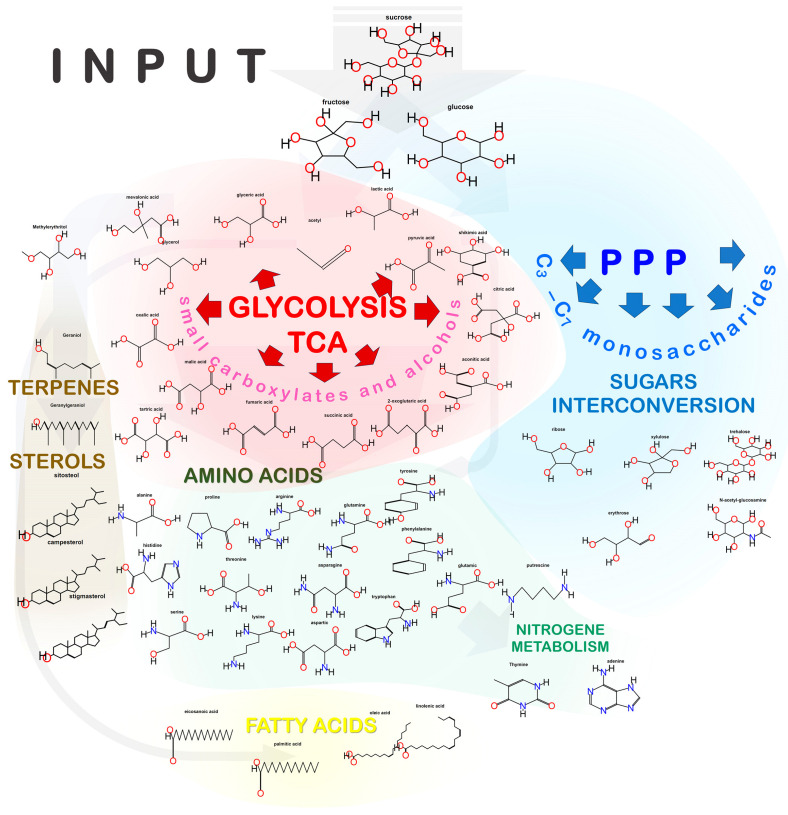
Machinery of biochemical diversity generation through sucrose metabolization in the heterotrophic plant cell.

Central (primary) metabolism is a term denoting a set of metabolic processes that provide a cell with carbon, energy, metabolites necessary for maintaining vital activity, and precursors for the synthesis of specialized (secondary) metabolites [56,57,58]. In heterotrophic cultures, the central metabolism is mainly aimed at the assimilation of organic substrates. They can be catabolized through glycolysis (Embden–Meyerhof–Parnas pathway), the tricarboxylic acid (TCA) cycle, and the pentose phosphate pathway (PPP) to produce energy. On the other hand, exogenous carbon can be incorporated into the metabolism and directed to the synthesis of amino acids, fatty acids, or other metabolites (Figure 1). In addition, incoming carbon can be deposited in the form of starch or lipids, and be mobilized by cells later after the depletion of external sources [58,59,60,61,62,63].

Thus, the analysis of the relationship between organic substrate uptake and the pathways of the central metabolism can shed light on fundamental life processes. This is needed for an elucidation of the mechanisms controlling the metabolism to improve productivity. Unveiling the details of various substrate assimilations could provide opportunities to overcome the limitations of biotechnological industries caused by the application of “expensive” substrates, such as sucrose. Alternatively, industrial waste could be used, as in the case of microalgae cultivation [64,65]. In this review, we will discuss the variety of organic substrates used to maintain plant cell cultures’ growth, and the main mechanisms by which these compounds are incorporated into metabolism. Since cell cultures are dynamically developing systems, special attention will be paid to possible changes in the assimilation of substrates at different stages of the cell cycle, including the differentiation and aging stages, as well as under a number of stress factors.

## 2. Heterotrophic Culture Specificity

According to the conditions of maintenance cultures, they can be divided into heterotrophic [31], mixotrophic [66], and autotrophic [67]. For heterotrophic cultures, the only source of carbon and energy is exogenous organic compounds. Photoautotrophic cultures receive light, but not an organic substrate. Mixotrophic cultures receive both organic substrate and light. In the last case, the conditions should not be confused with the actual trophic status of the cells. Mixotrophic cultures demonstrate a similarity to heterotrophic ones in terms of substrate availability in the medium. This can be expected due to the negative feedback loop suppressing photosynthesis during the accumulation of sugars [68,69]. So, potato plants growing on a medium with sugars obtained less than half of the carbon from photosynthesis, and just a tenth when sucrose concentration reached 8% [70]. One more argument for low photosynthetic impact under mixotrophic conditions is that the transition of *A. thaliana* cultures from mixotrophic to heterotrophic conditions did cause a slight increase in biomass growth rate, and not a decrease. The revealed increase in growth may be a result of a mitigation of damage from reactive oxygen species generated during photosynthesis [71].

Metabolic fluxes in plant cells vary intensively due to the presence of illumination [5]. Thereby photosynthetic and heterotrophic cell cultures are supposed to differ significantly in the spectrum of implemented metabolic reactions. Heterotrophic cell cultures are characterized by a large number of reactions associated with the exchange of sucrose and starch, fructose, and mannose, as well as inositol. At the same time, cultures are inferior in terms of reactions involved in fatty acid metabolism relative to the native heterotrophic organs. Darkness conditions causes a more developed ascorbate metabolism. Finally, the culture metabolome is sufficiently similar to the root metabolome rather than the metabolomes of other organs, which is expected due to the lack of photosynthesis [72]. 

The ability to assimilate an exogenous substrate as a preferred carbon source is one of the most important properties of cells in heterotrophic conditions. The transfer of suspension cultures to heterotrophic conditions can lead to the loss of chlorophyll and a decrease in photosynthetic activity [71,73,74]. Moving bleached cultures grown in darkness under light, at times, induces the restoration of the photosynthetic apparatus. However, the recovery of photosynthetic activity is not always possible [74]. Some heterotrophic cultures, such as BY-2, have completely lost the ability to photosynthesize [75]. In this case, the growth of cell cultures would be strictly limited by the amount and composition of the substrate [76].

It is obvious that a change in the trophic status of cells leads to complex metabolic adjustments. However, the systemic adaptation of higher plant cells’ trophic regime to heterotrophic culture has not yet been sufficiently studied. Initial data on the effect of light on the pattern of the accumulation of metabolites in *A. thaliana* cultures mainly concern secondary metabolites [77]. A greater accumulation of flavonoids, as well as a higher expression of genes encoding enzymes of its synthesis, was found under mixotrophic conditions compared to heterotrophic ones. Nevertheless, just a slight difference was revealed in the metabolite profiles of *A. thaliana* suspension cultures maintained in the darkness or under illumination [78]. Further analysis succeeded in discovering differences in the proteome [71] of *A. thaliana* cultures. In particular, the accumulation of chloroplast proteins was suppressed in the dark. At the same time, cell wall proteins were upregulated. The expression of enzymes associated with the central metabolism also changed [71]. 

Adaptation to trophic conditions affects lipid metabolism. Photosynthetic plants and cell cultures are characterized by a relatively high content of monogalactosyldiacylglycerols, digalactosyldiacylglycerols, sulfoquinovosyldiacylglycerols, and diacylglycerophosphoglycerols [79], while heterotrophic suspension cultures of *Chenopodium rubrum* demonstrate a lower level of linolenic acid. Generally, this fatty acid prevails in monogalactosyldiacylglycerols and digalactosyldiacylglycerols. Under heterotrophic conditions, the level of sterols increased in comparison with autotrophic cultures and leaves. Heterotrophic cultures were inferior to autotrophic cultures in terms of major phospholipid content and fatty acid spectrum [80].

## 3. Organic Substrates

### 3.1. Mobile Oligosaccharides

The repertoire of organic compounds capable of supporting the growth of plant cell cultures is restricted. These include metabolites that are easily transported between individual cells and various plant organs. A number of traits inherent in transported molecules, such as water solubility, neutrality, weak dissociation, chemical inertness, and lack of toxicity in high concentrations, are also used to characterize organic substrates for cultivation. 

#### 3.1.1. Sucrose

Sucrose is the major transport form of assimilated carbon and energy in higher plants [81,82]. Perhaps its properties allow it to be easily metabolized and therefore widely used as a substrate [45,66,83]. Cell cultures obtained from plant organisms of various systematic positions—both gymnosperms [73] and numerous angiosperms—have the ability to grow on sucrose-containing medium. These include cell cultures obtained for both monocotyledonous and dicotyledonous [45,66,84,85] plants, including such model objects as *A. thaliana* [85,86] and *N. tabacum* [15]. 

In higher multicellular plants, sucrose is transported through the phloem and reaches sink cells/tissues, where this metabolite is included in carbohydrate metabolism as a carbon/energy donor, or is stored by the cell. Sucrose uptake into the sink cell is provided by several transport systems, including protein SUTs (sucrose transporters), MSTs (monosaccharide transporters), and SWEETs (sugars will eventually be exported transporters). Along with this, sucrose’s irreversible hydrolyzation into glucose and fructose by apoplast invertases and a subsequent uptake of reaction products may also occur [87,88,89,90]. Plant invertases are divided into three types according to their optimum pH and localization in the cell. Acidic invertase is localized in vacuoles and neutral/alkaline in the cytosol and apoplast [91,92]. Sucrose synthase catalyzes the reversible cleavage of sucrose using UDP yielding fructose and UDP-glucose, which is known as a precursor in the synthesis of a number of metabolites [93]. Thus, an important question arises: what is the ratio of different pathways of sucrose utilization, and how does it change during culture development?

Fluxes modeling for heterotrophic *A. thaliana* cultures have shown that invertases provide the main pathway for sucrose incorporation in the metabolism [63]. For some cultures, including BY-2, the accumulation of glucose and fructose in the medium was accompanied by a decrease in the sucrose pool, which may also indicate the activity of extracellular invertases [94]. At the same time, the ability of the medium itself to hydrolyze sucrose is observed; this rises during the period of biomass accumulation and reaches the maximum level when growth is arrested. It is noted that the most intensive sucrose consumption precedes those of glucose and fructose, thus indicating that sucrose hydrolysis is prime and is followed by the absorption of its products [95]. The represented data are consistent with the fact that BY-2 cells secreted proteins into the medium and also acidify it, especially during the lag and log phases of culture development [96,97]. The composition of the secreted saccharides is quite complex and mainly consists of neutral sugars, including glucose and other hexoses [96], which may lead us to incorrectly overestimate the role of extracellular invertases in sucrose metabolism. There is an assumption that the culture medium is an active biochemical system which might be considered as an extracellular compartment [98]. The results of in vivo studies pointed out that the methods of sucrose metabolization in heterotrophic cells depend on the type of organ, tissue, and organism [87,88,99]. In the case of tomato fruit formation, it was found that during cell division, vacuolar acid invertase plays a major role in sucrose hydrolysis. Thus, the vacuole is thought to be responsible for supplying young dividing cells with carbon [100,101,102,103]. The conclusion is that several pathways are responsible for the sucrose incorporation in the metabolism, which provides maximum flexibility and efficiency for plant cells. Heterotrophic cell cultures are characterized by the participation of various invertases during development, and the intensity of this effect depends on the stage of the life cycle, while its integration with other pathways in the heterotrophic metabolism requires further investigation. Continuous reversible degradation and the synthesis of sucrose, known as the sucrose cycle, is often observed in heterotrophic plant tissues such as tubers, fruits, and seeds. It plays an important role in carbon portioning [91,104,105]. The cycle is of particular importance when sucrose efflux is impaired [106]. A possibility of sucrose turnover was also noted for cell cultures [107]. But, sucrose recycling is an energy-consuming process. Calculated energy costs vary up to 2/3 of ATP content. However, such high values are criticized and possibly overestimated [108].

#### 3.1.2. Raffinose

Members of the raffinose family oligosaccharides (RFOs) are another type of carbon transport form [82,109,110]. These are oligosaccharides which, like sucrose, include glucose and fructose residues with galactose residue addition. Raffinose plays an important role in various processes such as stress resistance and seed germination [111,112]. Raffinose-like sugars are considered as an important transport form of carbon for some plants, in particular from the families *Cucurbitaceae* and *Scrophulariaceae* [88,113]. A study of the composition of phloem sap showed the presence of oligosaccharides, but in smaller quantities than sucrose [110,114,115,116]. The role of raffinose in the redistribution of carbon in the plant has been confirmed by methods of genetic engineering [110]. The advantages of raffinose and other RFOs for maintaining the growth of cell cultures may be mediated by the ability to increase the concentration of organic carbon without elevating the molar concentration and, consequently, osmotic pressure [82]. For raffinose, the capacity to support growth at a level close to sucrose was shown in an analysis of heterotrophic tobacco cell culture growth [117], and of cell cultures obtained from some other plants such as wild carrot [118] and sugarcane [119]. RFO entry into the metabolism starts from its cleavage to sucrose and galactose, or other hexoses [82,111].

### 3.2. Sugar Alcohols

Sugar alcohols represent another group of metabolites identified to possess the transport function [82,120]. They can also play the role of reserves [121] and contribute to stress resistance [122]. A significant number of these were found in phloem exudates [114,115,116,123,124]. However, cell lines of only a few species are capable of utilizing sugar alcohols for growth supply. Usually, the growth rate of cultures using mannitol as a substrate is reduced compared to ones with sucrose or glucose [84,125,126]. Nevertheless, *Apium graveolens* cultures grow on medium with mannitol as quickly as with sucrose [127]. Sugar alcohols enter the metabolism through their conversion by dehydrogenases and oxidoreductases into hexoses [82,120,128]. Inositol, which is cyclic polyol, has been detected in the phloem of many plants [82,114,116]. It plays an important role in many processes [129]. However, attempts to maintain culture on a medium with inositol as the only carbon source were insufficient [130]. *Myo*-inositol is required as a component of MS media [15]. A slight growth stimulation with the addition of *myo*-inositol was observed when it was added to a sucrose-containing medium in the case of heterotrophic tobacco cultures [131].

### 3.3. Starch

The diurnal light cycle results in a dynamic interconversion of the various sugars observed in plant cells, including the accumulation and degradation of starch [5,132]. The multiplex alteration in starch content is also noted for fruits in the formation and ripening stages. Fruiting initiation is accompanied by starch synthesis, which provides sink power for sucrose transport. At the end stage, on the contrary, starch degradation compensates for the weakening of the phloem transport function [101].

An analysis of heterotrophic cell culture growth showed that starch can also serve as a substrate, but with a lower efficiency than that of sucrose and major hexoses [84,133,134]. Some plant cells are able to assimilate products of starch degradation, including maltose, a dimer of α-glucose. In general, the growth intensity of this substrate is less than that of sucrose or glucose [84,117,133,135]. However, in the case of *Catharanthus roseus*, maltose provided growth at a level close to glucose [136]. The ability to grow on media with dextrin has been demonstrated for some callus cultures [85,137]. Probably, the incorporation of these compounds in the metabolism of cells is based on gradual hydrolysis to obtain low-molecular sugars.

### 3.4. Other Complex Sugars

Another massive cellular compartment which could be a carbon source is the cell wall. The mechanism underlying the application of cell wall components as substrates for culture growth remains poorly understood. Cellobiose (β-glucose dimer) is a product of cell wall polymer hydrolysis. Callus cultures vary greatly in their ability to grow on a medium with this disaccharide. The heterotrophic culture of tissues obtained from marigold demonstrated almost the same growth intensity as on sucrose, while tissues of several other species grew much less actively, if at all [137]. Suspension cultures of sugarcane cells also showed growth on cellobiose at only slightly lower rates than on sucrose [119]. Some data indicated the maintenance of cultures on medium with pectins [137]. Thus, the potential of plant cells to assimilate to oligosaccharides originating from the cell wall is probably high. The importance of oligosaccharides is indirectly evidenced by the fact that even lactose can also be utilized by plant cell cultures [84,117,119,138]. This dimer of glucose and galactose is known primarily as a component of milk, and its presence in plants has been questioned for a long time [139].

### 3.5. Monosaccharides

#### 3.5.1. Fructose and Glucose

The metabolism of oligo- and polysaccharides leads to the formation of hexoses, the most common of which are fructose and glucose. Therefore, it is not surprising that these two hexoses are also perfectly absorbed by the cells of many plants [125,133,140], including tobacco [126,135,141,142,143] and *A. thaliana* [61,144,145]. In some cases, the combined presence of both of these sugars in the incubation medium gave a better biomass growth compared to those with their individual use [84]. But, some results found the opposite [130], or there were no differences to growth under individual monosaccharide application [146]. In addition, glucose and fructose are not completely equivalent substrates. Glucose provided higher productivity in some cases [125,140]. If both of these sugars are present in the medium, sometimes glucose is preferably absorbed [146]. In one study, only glucose was absorbed by cells, which triggered fructose accumulation in the medium [147]. Therefore, it is considered that glucose is a preferable substrate.

Sucrose synthase activity results in UDP-glucose formation, which is an activated glucose moiety in the synthesis of various polysaccharides, glycolipids, and secondary metabolites [148]. Glucose metabolism begins with phosphorylation by hexokinase. In a similar way, fructose and other hexoses enter the metabolism. Of the several known pathways for glucose cleavage, only two pathways appear to be major pathways in plants: glycolysis and PPP. However, a fluxomic analysis of heterotrophic cells showed that glucose is catabolized mostly through glycolysis and the TCA cycle, while the contribution of PPP was much smaller [61,105]. 

#### 3.5.2. Other Monosaccharides

The metabolic analysis of various plant organs and tissues, as well as phloem sap [114,115,116,123,124], detected wide spectra of sugars and their derivatives. The intensive sugar metabolism in plant cells is a basis for groups of monosaccharides in maintaining cell growth. Galactose is another representative of hexoses. Its residue, as mentioned above, is part of the RFO. But, growth rates with this compound as a carbon nutrient are often much slower than with sucrose [84,133,136,149,150]. For example, tobacco cells usually cannot grow on media containing galactose [117,135]. Alternatively, the growth is observed after a longer lag phase [95]. The metabolism of galactose begins with its phosphorylation and subsequent conversion into glucose phosphate, which enters in glycolysis. In another variant, first UDP-galactose is formed from galactose-1-phosphate, and then UDP-glucose. Insufficient galactose-1-phosphate conversion may lead to a depletion in the phosphate pool, which is considered a reason for the negative effect of galactose on cell culture growth [151]. Plant cells vary greatly in their ability to assimilate mannose. The cell lines of wild carrot and ipomoea were characterized by intensive growth on a medium with mannose. They were comparable with results obtained on media with glucose and fructose, but were less comparable with sucrose. Mixotrophic cultures of *A. graveolens* showed the same growth on mannose as on sucrose [127], whereas cannabis cell culture grew three times slower [84]. Similarly, cells of other species showed less growth on mannose [134,150]. Weak growth in the presence of mannose was detected for wild-type tobacco cell lines [141]. The key enzyme of mannose assimilation is mannose-6-phosphate isomerase (PMI). It converts mannose-6-phosphate into fructose-6-phosphate, which is destined for glycolysis. Usually, PMI is expressed at a very low level. This limits the growth of plant cultures on mannose-containing substrate. The key role of PMI is confirmed by the fact that a mutant *N. tabacum* line with an increased growth rate on mannose was characterized by an increased level of the activity of this enzyme and corresponding gene expression [141].

Testing the ability of a number of pentoses to support culture growth has shown them to be very limited [84,119,150]. Callus cultures of tobacco cells were unable to grow on media containing both xylose and xylitol. However, weak growth was possible on a medium with ribose [135]. The mechanism of pentose metabolism also begins with phosphorylation and involvement in PPP reactions. Nevertheless, this pathway may be severely limited for several reasons: firstly, the inhibitory effect of the accumulation of free non-phosphorylated pentoses; secondly, the rather low rate and intensity of phosphorylation; and thirdly, the low activity of PPP in heterotrophic cultures.

### 3.6. Glycerol

Glycerolipids, especially triacylglycerides, represent another group of compounds that can be accumulated by plants as storage compounds. They are insoluble in water, which limits their use as suspension culture substrates. Nevertheless, the potential of cell culture to grow on a medium with glycerin has been well investigated. It is quite surprising that cultures of many plant species grow well on a medium with glycerin: *Cynara cardunculus* [133] *Daucus carota*, *Cannabis sativa*, and *Ipomoea* sp. [84] are among them. *Citrus suhuiensis* cell culture grew on glycerol, but weakly compared to sucrose and hexoses [140]. Wild-type *N. tabacum* cell cultures were completely incapable of glycerol utilization However, a mutant was obtained on which callus cultures acquired the ability to grow weakly on a medium with glycerol [135]. Other C_2_-C_4_ alcohols, apparently, are also unable to support the growth of plant tissue culture, and may even be toxic. However, there are a few exceptions. *C. roseus* tissues were characterized by poor growth on media not only with glycerol, but also methanol, ethanol, and butanol as carbon sources [152].

The entry into the metabolism for glycerol begins with its ATP-dependent phosphorylation by glycerol kinase to form glycerol-3-phosphate (G3P) [153]. Spraying tobacco leaves with glycerol has been shown to increase the expression of glycerol kinase [154]. When glycerol is added to starving root tips, the G3P content elevates, and the ATP pool decreases dramatically [155]. G3P can be directed to the synthesis of glycerolipids. Another scenario is glycerol’s conversion by G3P dehydrogenase to dihydroxyacetone phosphate (DGAP), which might be directed up the gluconeogenic pathway for the synthesis of sugars [153]. Interestingly, the cultures of *N. tabacum* mutant, which is capable of poorly assimilating glycerol, are not able to grow on media with G3P or dihydroxyacetone [135]. Otherwise, DGAP can be converted to pyruvate during glycolysis and sent to the TCA cycle [153]. The addition of glycerol and dihydroxyacetone can reduce the decrease in the activity of phosphoenolpyruvate carboxylase and pyruvate kinase, as observed under starvation in the cells of maize root tips [155].

Glycerol application to starving sycamore cells does not eliminate the effects of starvation, such as drops in the levels of sucrose, starch, and hexose phosphates. Nevertheless, an increase in G3P in the cytoplasm has been observed. Sycamore cells are able to survive for more than three weeks on a medium with only this triol. Glycerol competitively inhibits glucose-6-phosphate isomerase, which stops PPP. Glycerol then becomes the only resource of carbon for respiration and is not used for synthesis. But glycerol supports dissociated respiration well [156]. When maize root tips are starved, exogenous glycerol and dihydroxyacetone are able to supply TCA with carbon. However, these substrates are not able to support growth [155]. Spraying tobacco with glycerol led to changes in the metabolome and transcriptome. The content of amino acids, sugars, and carboxylates increased. The glucose level lifted especially strongly. Transcriptomic analysis showed that carbohydrate metabolism, including sucrose synthesis, was activated, as was nitrate assimilation [154].

### 3.7. Carbonic Acids

Like other aerobic organisms, plant cells are characterized by respiration. For heterotrophic cells, this is the main way to obtain energy. In the profiles of the metabolites of plant cell cultures, various carbonic acids including intermediates of glycolysis and TCA are detected [157,158,159,160], possibly due to the accumulation of various carboxylic acids in the central vacuole [161,162,163]. Nevertheless, plant cells are significantly limited in their ability to assimilate carboxylic acid as the only carbon source. Very weak growth was demonstrated for several callus cultures on medium with TCA intermediates of succinate, glutarate, and fumarate [152]. Pyruvate, a key metabolite linking glycolysis and the TCA cycle, could not support the growth of tobacco cultures in comparison to dihydroxyacetone, another intermediate of glycolysis [135]. Simultaneous presence in the medium of some carboxylates, including pyruvate and TCA cycle intermediates, along with sucrose (the main substrate), stimulates the growth of suspension cultures of tobacco cells [164] and some other cultures [137]. Succinate has a particularly strong effect. It is absorbed during the growth of the culture and its labeled carbon is incorporated into other metabolites. In addition, acids not directly related to the activity of TCA, such as lactate, acetate, tartrate, and glycolate, have a weak effect on growth intensity [164].

### 3.8. Amino Acids

Under stress, starvation, and aging, the cell can mobilize carbon not only from specialized reserve molecules, but also from other cellular resources. One of the processes that provides the degradation of cellular structures is proteolysis. The amino acids that are formed as a result of it can be anabolized or/and catabolized directly, or can be converted into transport forms and directed to other organs and tissues. In addition, plants are able to use proteins as specialized reserves of nitrogen and carbon accumulated into protein storage vacuoles [165,166]. Free amino acids are widely represented in the profiles of cell culture metabolites, and some of them are in relatively high concentration [157,158,167]. These metabolites are an important component of phloem sap, and in some cases their total concentration can be comparable to that of sucrose [114,123,168,169]. Interestingly, some amino acids, especially glutamine, are a form of nitrogen transport in plants [170]. Glutamine is often characterized as having the highest content in phloem sap [168,171,172]. Moreover, plants have the ability to uptake organic nitrogen from the soil [13,173,174]. Some amino acids, in particular L-glutamine and L-asparagine, promote the growth of *A. thaliana* seedlings [174]. However, the addition of amino acids, regardless of their proteinogenic properties, to the culture medium did not stimulate the growth of *N. tabacum* cell cultures [169].

**Figure 2:**
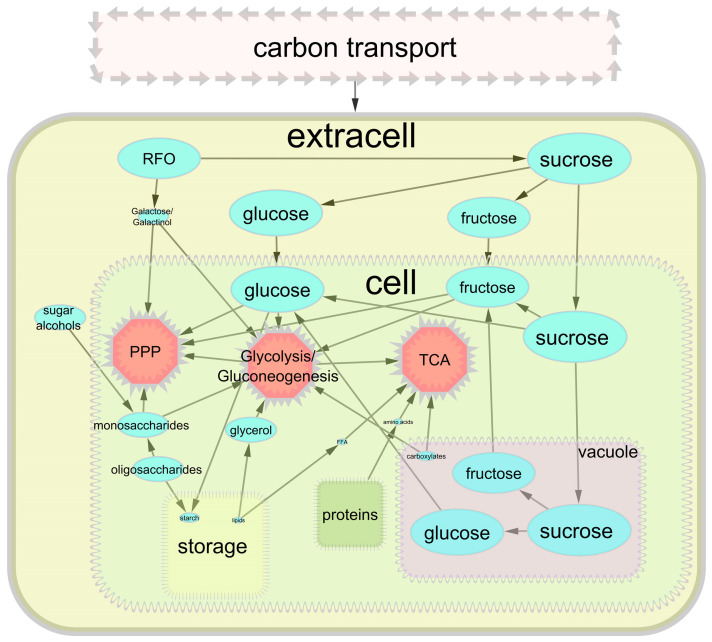
Heterotrophic sources of carbon and their capability (size of the ellipses) to maintain plant cell growth.

Thus, a broad spectrum of metabolites, mainly carbohydrates, can be used as substrates capable of supporting the growth and development of cell cultures of higher plants in heterotrophic conditions. A significant role is played by metabolites that represent a transport form, for example, sucrose and its decomposition products (Figure 2).

## 4. Alterations in Culture Metabolism over Time

### 4.1. Development 

Cell cultures are dynamic systems where the state of the medium and cells are cross-regulated. The pattern of the medium changes is a result of the constant consumption of its components, as well as a result of the excretion of metabolism products and enzymes by cells. Simultaneously, the growth and development of cells can change their metabolic requirements, and, consequently, alter the intensity of uptake of various components from the medium [97,175,176,177,178]. 

Some of the changes in culture cells are similar to those that occur during cell specialization in plant tissues in vivo. The processes of specialization/differentiation are of great importance for the absorption and distribution of resources in the cells of multicellular organisms. Systematic biological studies have revealed the essential specificity of different organs and tissues of plants, which they acquire during their development [179]. Cytological and biochemical processes in culture cells storing starch and the cells of the root cap are similar [20]. The fact that this process may be similar to the one going on in the whole organism is indicated by the fact that, in culture, this specialization is regulated by phytohormones. For example, an excess of sucrose that occurs after the division cessation is not sufficient enough to initiate the accumulation of starch. This process requires a decrease in auxin levels [180].

### 4.2. Expansion Growth

One of the prominent examples of plant cell specialization/differentiation is expansion growth, representing a rapid increase in cellular volume. This process underlies both plant growth and the accumulation of storage substances in specialized organs. A specialized form of expansion is elongation growth. Elongation growth is a rare process in suspension cultures. *N. tabacum* BY-2 culture is one of few which show this trait [23]. Most research is aimed at studying the mechanisms that regulate the properties of cell walls. The mechanisms underlying the changes in the metabolism of cells undergoing elongation growth, or other processes of differentiation, remain poorly understood.

One of the most important events during osmotic expansion growth is the accumulation of osmotic compounds, the main group of which are sugars. The osmotic pressure created in the vacuole remains consistently high throughout cell development. The main source of sucrose in vacuoles is importation [103]. Models have shown that exogenous substrates consumed during the enlargement of tomato pericarp cells are important for the accumulation of osmotic compounds in vacuoles [101,181]. During tomato fruit pericarp cells’ transition to expansion, the sucrose flow through acid invertases decreases and the role of sucrose synthase and neutral invertases increases [100,103]. Simultaneously, changes in the activity of glycolysis occur. Expansion is associated with a high activity of enzymes associated primarily with the lower part of glycolysis [100], whereas the activity in the upper part of glycolysis may decrease [100,103]. Meanwhile, the contents of sucrose and glucose-6-phosphate are dramatically reduced. As a result, the activity of starch metabolism hexose levels increases [100]. Carbohydrate metabolism is closely related to energy metabolism. It is always a balance between the availability of substrates for the ATP supply of cellular processes and the necessity of sufficient energy required for the subsequent conversion of carbohydrates. Additional ATP consumption during expansion growth is necessary for the function of the H^+^-ATPases of both the plasmalemma and tonoplast. In tomato pericarp cells, elongation growth is comparable to cell division in culture in terms of ATP consumption, but its budget is different. Actively dividing cells of tomato suspension cultures are characterized by a higher level of carbon flux in the synthesis of cellulose, protein, starch, and lipids. For pericarp expansion cells, osmotic compound accumulation is a major cost item [101,105]. One of the groups of osmolytes in the vacuoles is carboxylates. Their accumulation plays a role during the period of active proliferation [103]. Further on, during expansion, the content of malate and citrate decreases, and the activity of TCA enzymes becomes low [103]. Nevertheless, some data indicate that the level of citrate and malate increases slightly during expansion and there is some activation of carboxylate metabolism in the mitochondria and their subsequent export to the vacuole [101,102].

### 4.3. Aging

Depletion, and in some cases intoxication of the medium, can cause a slowdown in the growth and process related to senescence. During the transition of BY-2 cultures to the stationary phase, the number of mitochondria and peroxisomes decreases. The representation of plastids does not change or increase slightly, but organelles lengthen and accumulate starch grains [182]. At this stage, the degradation of the Golgi apparatus has been noted [183]. In addition, peroxisome number decreases during culture development [184]. Metabolic rearrangements are indirectly indicated by the results of the transcriptome analysis of BY-2 cells in the lag, log, and stationary phases. For example, the expression of a large number of genes involved in cell division was downregulated after the transition to the stationary phase. Genes of the lipid metabolism were activated at the early phases or at the stationary phase, reflecting the normal dynamics of membrane formation and storage lipid accumulation [177].

Changes during culture ageing are also manifested at the metabolomic level. For example, complex dynamics of the metabolome were noted for mixotrophic suspension poppy cultures. A decrease in the level of sucrose and malate over time and the accumulation of some amino acids was estimated [27]. The study of heterotrophic tomato cell cultures revealed the accumulation of organic acids (malate and citrate) at the end of the growth phase, but the amount was relatively small. Comparatively, after the end of the growth phase sucrose began to accumulate [105]. Such dynamics are similar to the changes in cells during apple fruit ripening: the content of organic acids, most amino acids, sugar phosphates, and glucose decreased, but sucrose and fructose accumulated [161]. The development of cell cultures is accompanied by changes in the accumulation of nitrogen metabolism intermediates. During the period of active growth of BY-2, the content of free and soluble conjugates of amines—spermine and spermidine—in cells increased, and the content of putrescine decreased. A shift in the activity of polyamine synthesis enzymes was detected [185].

The ageing of cultures may be linked to an alteration in the balance of various methods of energy supply. The primary metabolism depends on the uptake of exogenous substrate and generally maintains structural stability, even during depletion by growth termination and transition to the stationary phase. The end of heterotrophic tomato cell proliferation in culture coincided with a drop in flux through glycolysis and TCA and the possibility that it might turn back then to increase again [105]. It has been shown that with the ageing of heterotrophic tobacco cell cultures, the activity of aerobic respiration decreases [186]. This phenomenon, together with the increase in glycolysis in the late stages of development, are supposed to be mechanisms of oxidative stress reduction and slow down the aging process [187].

The growth rate of culture and metabolic activity are closely related to the composition of membranes and, consequently, the spectra of fatty acids and their desaturation. The young suspension culture of *C. sativa* was characterized by a higher level of unsaturated fatty acids, while old ones were higher saturated and contained aromatic compounds [25]. An analysis of the growth rate of *A. thaliana* suspension cultures and the composition of unsaturated fatty acids showed that there was a close relationship between these parameters [188]. 

The senescence of plant organisms and their organs is a strictly programmed process, and because of that it is not fully reproduced in cell culture. Old starving heterotrophic cultures of *A. thaliana* grown on sucrose are quite different from natively ageing plant organs. This was confirmed by modifications in the gene expression profiles [189]. The aging of plant cell cultures was accompanied by the exhaustion of sugars in the medium [190,191]. A decrease in sugar content is a common reaction to the carbohydrate starvation of cells in culture [192,193,194,195]. The transition to the stationary phase resulted in a decrease in carbon flow into the synthesis of amino acids, as in the case of heterotrophic tomato cell cultures [105]. On the other hand, starving *A. thaliana* suspension cultures demonstrated an elevation in malate and amino acid content [193]. Also, the senescence of organs may be accompanied by sugars and some increases amino acids as a part of the process of complex molecule destruction within the remobilization of carbon and nitrogen [8].

## 5. Carbon Starvation

In heterotrophic cultures, cell nutrition is completely dependent on an external carbon source. This, in turn, closely links the productivity of the culture with the amount of substrate. Often, the duration of the log phase and the final density of the culture positively depend on the initial concentration of the substrate [47,125,196]. But this dependence is limited, and there is usually a substrate concentration that ensures maximum culture growth, which depends on the type of culture and the substrate [152,197]. One of the reasons for the growth wane with substrate elevation is the increase in osmolarity [198,199]. It should also be noted that the consumption of substrates causes a decline in osmolarity during growth [126]. This requires additional adaptation costs. In addition, a variation in osmolarity can induce morphogenetic effects, including the initiation of somatic embryogenesis [198].

It is obvious that, during culture growth, the substrate is depleted and starvation starts. The process of starvation is characteristic not only of cell cultures but also of cells within native organs when the photosynthetic activity of source tissues is reduced or when transport systems are disturbed, especially under stress conditions [132]. The capacity to control the media composition makes cell cultures a convenient model system to study physiological, cytological, and metabolic changes under starvation [200].

There are three stages of starvation that could be distinguished [201]. The first one, acclimation, is characterized by a shortage in the level of respiration following storage mobilization. During the survival phase, an intense catabolism of proteins and lipids occurs. The third phase of disorganization is irreversible. The physiological activities of all processes in this period decrease, and cells die [201].

Usually, the removal of sucrose from media leads to a rapid decrease in the level of respiration [194,202,203]. In the first dozen minutes, a slight decrease in the level of respiration in sycamore cell cultures was accompanied by an intensive decrement in the content of sugar phosphates—not only sucrose, but also fructose, glucose, and UDP-glucose—while the intensity of uncoupled respiration did not slow down, indicating that respiratory limitation was not mediated by substrate availability. The probable cause is a general reduction in metabolic activity and insufficient energy resources for the phosphorylation of sugars. Presumably, this is due to a limitation in the intensity of glycolysis, since the drop in glucose-6-phosphate is observed only in the cytosol, and not in plastids. A decrease in the content of sugar phosphates leads to the accumulation of nucleotide triphosphates [195]. With longer starvation (more than 20 h), the internal reserves of sucrose and starch become depleted, which triggers mitigation in both normal and uncoupled respiration. The lessening in respiration is at least partially due to a decrease in the number of mitochondria and is apparently not related to the availability of substrates. After the new addition of sucrose, respiration and the pools of sugar phosphates are restored. The degree of this recovery depends on the supply of Pi [192,194]. In rice cell cultures, sucrose starvation for two days leads to the repression of genes encoding glycolysis enzymes (with the exception of hexokinase), as well as PPP, TCA, and oxidative phosphorylation enzymes. As a result, the pathways associated with the development of other sources of energy and carbon are activated [204]. In BY-2 cell cultures, starvation induces peroxisome degradation and, probably, catabolic process repression [184]. The starvation of *A. thaliana* cells is accompanied by a downregulation in the expression of genes involved in protein synthesis, cell division, and energy metabolism [202]. 

During cell starvation, a mobilization of carbohydrate reserves is observed. Upon the general repression of starch and sucrose metabolism genes, some genes associated with starch hydrolysis were found to start the expression intensively. Starvation elevates the expression level of genes associated with trehalose metabolism. This glucose dimer is considered an alternative carbon source in the case of sucrose deficiency [202,204]. Starvation also leads to an increase in the level of transcripts of galactosidases and glycosylhydrolases. Sugar transporter genes, both sucrose and monosaccharide, were characterized by increased expression during the first 12 h of starvation in rice cells, which may also be important for the redistribution of carbohydrates in the cell [204]. It should be noted that sugar starvation induces photosynthetic genes in *A. thaliana* suspension cells [202].

Vacuolar pools of sucrose and malate are probably a fast source of carbon and energy. But, they are not able to maintain metabolic processes at the same level. The reason may be insufficient transport activity through the tonoplast from the vacuole [195]. From gluconeogenesis, organic acids can be transformed into sugars [205]. Utilization pools of carboxylates as carbon for sugar synthesis could be taken from the upregulation of phosphoenolpyruvate carboxykinase under starvation in rice cells [204]. In cucumber suspension cells, the expression of genes encoding two marker enzymes of the glyoxylate cycle (isocitrate lyase and malate synthase) was induced with sucrose, mannose, and fructose deficiency in the medium [206]. An increase in the level of malate accompanied the starvation of *A. thaliana* cells. At the same time, the level of other TCA intermediates did not change much [193]. 

Autophagy is a catabolic process strictly regulated at the molecular genetic level [207]. It is observed during starvation, stress, senescence, and some developmental stages. Autophagy plays a key role in the mobilization of reserves and, as a consequence, in the resistance of cells to stress factors [208,209,210,211,212]. Autophagy during the starvation of cell cultures, including *A. thaliana* [213,214] and *N. tabacum* [184,215,216,217], is studied intensively. It has been shown that cell starvation is characterized by high proteolytic activity. It is followed by an increase in the level of free amino acids, as, for example, in the case of the suspension cells of *A thaliana* [193]. The pool of amino acids, especially branched ones, is disposed of as respiratory substrates, or can be deaminated and sent to gluconeogenesis. This is indicated by an increase in the level of expression of genes of enzymes associated with branched amino acid metabolism [202,204]. The catabolism of amino acids plays a particularly important role in supplying cells with energy during starvation or stress. The activation of these pathways distinguishes starving ageing *A. thaliana* cultures from developmental ageing leaves and brings them closer to darkness-induced senescence [202]. 

On the other hand, it has been shown that starvation also includes the degradation of membranes. In BY-2 cultures, a decrease in the amount of phospholipids occurs under starvation. Moreover, this process is apparently not related to autophagy [218,219]. The starvation of suspension cultures of rice cells is followed by a downregulation of genes coding enzymes involved in the synthesis of fatty acids [204]. Genes encoding lipase and fatty acid β-oxidation enzymes are induced in suspension cultures of rice [204] and Arabidopsis [202]. The oxidation of fatty acids is accompanied by the generation of H_2_O_2_. Catalase is involved in the detoxification of H_2_O_2_ produced in glyoxysomes. A significant induction of the glyoxysomal catalase gene accompanies an increase in the expression of acyl-CoA oxidase when sucrose is removed from the suspension cultures of *A. thaliana* [220]. Culture starvation and leaf aging also induce fatty acid α-oxidation genes. Cell cultures differ from the ageing leaves in the activation of the expression of a larger number of lipid catabolism genes [202]. The importance of lipid oxidation and, possibly, gluconeogenesis, is also indicated by the fact that plastids, ribosomes, and EPR are destroyed earlier than mitochondria and peroxisomes. The consequence of the absence of sucrose for BY-2 cultures is the degradation of *trans*-Golgi network proteins, which is an adaptation of vesicle transport to starvation [221]. 

Unfortunately, resource mobilization cannot provide long-term vital activity to starving cultures, but rather precedes death. In addition, the irreversibility of the changes revealed at the final stage of starvation can be mediated precisely by the initiation of the autophagy process. In the case of suspension cultures of *A. thaliana*, after 24 h of starvation, cell viability begins to fall rapidly. After 48 h, the culture loses the ability to recover after replanting, which indicates the arrest of proliferation [202]. This effect might be related to the weak ability of cells to assimilate metabolic pools and products of lipid and protein degradation as carbon sources.

## 6. Conclusions and Future Perspectives

Summing up, the ability of plant cells to grow in heterotrophic cultures utilizing external organic substrates is an inherent trait formed during complex multicellular organism evolution. Heterotrophic cultures can be used as efficient models for investigations focused on the metabolic effects of various substrates. However, as the results of numerous experimental studies show, the spectrum of these organic compounds is limited compared to those assimilated by microalgae. There are several possible reasons for this restriction. Firstly, the chemical composition of the environment surrounding the cell inside a multicellular organism is less variable. Secondly, specialization limits the metabolic potential of a cell. Performing certain functions in the organism, a cell expresses only a part of its genome and modifies metabolic pathways associated with cell specialization. Cell specialization means that there is a necessity for carbon redistribution, and thus the appearance of universal transport forms. The other side of the coin is that cells have developed a sophisticated system for the precise uptake and metabolization of these molecules. From this point of view, sucrose is the most universal and effective molecule for plant cell cultivation. Raffinose and the products of hydrolysis, such as glucose and fructose, are also valuable carbon nutrients, but to a lesser extent. Other transported sugars, such as starch and its degradation products, are also able to support culture growth. Carbonic and amino acids are intensively accumulated and metabolized in plant cells, but are not as in demand as external carbon sources. Unfortunately, the reasons for these differences in substrates are still far from being understood. Probably, unlike transport forms, the intensity of the metabolism of these substrates varies greatly and depends on multiple factors such as dual light regimes, stages of development, stress responses, and cell specialization. In cell culture maintained under constant conditions, these mechanisms are perhaps not fully realized, and can be loosened. Cell cultures vary greatly in their ability to assimilate organic substansces, even at the strain level. Possible sources of this diversity include the traits the species cultures were isolated from, the partial specialization of cells, culture conditions, and genetic and epigenetic alteration during long-term cultivation. These variations need to be studied to enlarge the spectrum of molecules that could be used as substrates.

Another important issue is the existence of intensive futile metabolic cycles, which consume a significant portion energy. In cell cultures that are well supplied with substrate, the lack of activity associated with a reduction in cell function in turn causes an unintended abundance of energy and carbon. Therefore, futile cycles could be intensified and undesirable energy loss increased. This can reduce the resistance and productivity of cell cultures. In addition, the problem of the instability of cultures, including under starvation, can be explained by the lack of the self-sufficiency of the regulatory and metabolic systems in plant cells, since they depend on organismic signals. Cells separated from the organism do not provide the necessary storage, mobilization, or modulation for physiological activity.

Finally, carbon acquisition by plant cells in culture gives an opportunity for basic and biotechnological research. It seems appropriate to use “omic” technologies for the systematic analysis of the processes occurring in the dedifferentiated cells of calli and suspension cultures, and to identify differences from the processes occurring in individual cells of integral plant organisms.

## Data Availability

Not applicable.
